# High-performance presurgical differentiation of glioblastoma and metastasis by means of multiparametric neurite orientation dispersion and density imaging (NODDI) radiomics

**DOI:** 10.1007/s00330-024-10686-8

**Published:** 2024-03-15

**Authors:** Jie Bai, Mengyang He, Eryuan Gao, Guang Yang, Chengxiu Zhang, Hongxi Yang, Jie Dong, Xiaoyue Ma, Yufei Gao, Huiting Zhang, Xu Yan, Yong Zhang, Jingliang Cheng, Guohua Zhao

**Affiliations:** 1 Department of Magnetic Resonance Imaging, the First Affiliated Hospital of Zhengzhou University, Zhengzhou, 450052 China; 2 Henan Engineering Research Center of Medical Imaging Intelligent Diagnosis and Treatment, Zhengzhou, 450052 China; 3 School of Cyber Science and Engineering, Zhengzhou University, Zhengzhou, 450001 China; 4 Shanghai Key Laboratory of Magnetic Resonance, East China Normal University, Shanghai, 200062 China; 5 School of Information Engineering, North China University of Water Resources and Electric Power, Zhengzhou, 450046 China; 6 MR Research Collaboration, Siemens Healthineers, Wuhan, 201318 China

**Keywords:** Glioblastoma, Solitary brain metastasis, NODDI, Multiple volumes of interest, Deep learning

## Abstract

**Objectives:**

To evaluate the performance of multiparametric neurite orientation dispersion and density imaging (NODDI) radiomics in distinguishing between glioblastoma (Gb) and solitary brain metastasis (SBM).

**Materials and methods:**

In this retrospective study, NODDI images were curated from 109 patients with Gb (*n* = 57) or SBM (*n* = 52). Automatically segmented multiple volumes of interest (VOIs) encompassed the main tumor regions, including necrosis, solid tumor, and peritumoral edema. Radiomics features were extracted for each main tumor region, using three NODDI parameter maps. Radiomics models were developed based on these three NODDI parameter maps and their amalgamation to differentiate between Gb and SBM. Additionally, radiomics models were constructed based on morphological magnetic resonance imaging (MRI) and diffusion imaging (diffusion-weighted imaging [DWI]; diffusion tensor imaging [DTI]) for performance comparison.

**Results:**

The validation dataset results revealed that the performance of a single NODDI parameter map model was inferior to that of the combined NODDI model. In the necrotic regions, the combined NODDI radiomics model exhibited less than ideal discriminative capabilities (area under the receiver operating characteristic curve [AUC] = 0.701). For peritumoral edema regions, the combined NODDI radiomics model achieved a moderate level of discrimination (AUC = 0.820). Within the solid tumor regions, the combined NODDI radiomics model demonstrated superior performance (AUC = 0.904), surpassing the models of other VOIs. The comparison results demonstrated that the NODDI model was better than the DWI and DTI models, while those of the morphological MRI and NODDI models were similar.

**Conclusion:**

The NODDI radiomics model showed promising performance for preoperative discrimination between Gb and SBM.

**Clinical relevance statement:**

The NODDI radiomics model showed promising performance for preoperative discrimination between Gb and SBM, and radiomics features can be incorporated into the multidimensional phenotypic features that describe tumor heterogeneity.

**Key Points:**

• *The neurite orientation dispersion and density imaging (NODDI) radiomics model showed promising performance for preoperative discrimination between glioblastoma and solitary brain metastasis.*

• *Compared with other tumor volumes of interest, the NODDI radiomics model based on solid tumor regions performed best in distinguishing the two types of tumors.*

• *The performance of the single-parameter NODDI model was inferior to that of the combined-parameter NODDI model.*

**Supplementary Information:**

The online version contains supplementary material available at 10.1007/s00330-024-10686-8.

## Introduction

Glioblastoma (Gb) and solitary brain metastasis (SBM) are the most common brain tumors in adults [1–6]. Preoperative differentiation between Gb and SBM is clinically critical for aiding individualized treatment decisions. Histopathology is the gold standard for diagnosing Gb and SBM, usually with biopsy or open surgical resection [7]. However, biopsy or open surgical resection may increase the risk of morbidity and mortality in older or weak patients. Therefore, an accurate non-invasive diagnosis is preferable.

Magnetic resonance imaging (MRI) is the primary imaging modality for diagnosing brain tumors to obtain multi-view information to help neuroradiologists differentiate various pathologies. However, as both Gb and SBM often present a similar anatomic MRI appearance, morphological MRI is sometimes ambiguous in differentiating the two types of tumors [8]. Moreover, up to 40% of cases are incorrectly classified by morphological MRI alone [9]. Diffusion-weighted imaging (DWI) can be used to assess the spread and proliferation of brain tumors and can differentiate between Gb and SBM [10, 11]. Advanced neurite-oriented diffusion and densitometric imaging (NODDI) is an extension of DWI, which includes the isotropic volume fraction (ISOVF), intracellular volume fraction (ICVF), and orientation dispersion index (ODI) [12–16]. These parameters can assess the complexity and heterogeneity of the brain microstructure in vivo, and can also allow quantitative analysis to elucidate other disease pathologies. In a pioneering study of NODDI, the extracellular volume fraction (VEC) in the peritumoral signal change area was more useful than intracellular and isotropic volume fraction in distinguishing Gbs from SBMs [17]. Currently, VEC, calculated through the ISOVF and ICVF, can be replaced by the ODI. Another study assessed the NODDI histogram analysis for distinguishing between two tumor types and compared the diagnostic performance of placing regions of interest (ROIs) [18]. Traditional diffusion data analysis methods involve pixel/voxel comparisons to identify lesion differences or rely on the mean signal for ROI-based investigations. These traditional studies are often hypothesis-free and only reveal differences between Gb and SBM across sparse imaging features, providing insufficient information to elucidate the complex biology underlying the identified differences in diffusion signals.

Radiomics can acquire quantitative imaging signatures at a high throughput and correlate imaging signatures with targeted clinical outcomes [19–25]. ROIs and volumes of interest (VOIs) are delineated on the subregions of tumors and lesions [18, 26, 27]. Thus, radiomics offers diverse imaging information and helps to explore the tumor microenvironment by analyzing well-defined subregional features that more precisely describe tumor heterogeneity. Radiomics based on multiple ROIs/VOIs can help provide potential evidence for the correlation between imaging and tumor heterogeneity, facilitating the integration of advanced imaging techniques and analysis methods into clinical practice. Additionally, it has also been used to identify survival stratification in Gb [28].

To the best of our knowledge, no radiomics studies have been conducted based on NODDI to distinguish between Gb and SBM. In this study, we evaluated the utility of radiomics analysis of NODDI based on multiple VOIs in identifying SBM and Gb, compared its discriminatory performance with morphological MRI and diffusion MRI, and attempted to analyze the biological significance of the NODDI radiomics model.

## Materials and methods

### Ethics consideration

This retrospective study was approved by our institutional ethics committee, which waived the requirement for obtaining informed patient consent.

### Patients

The medical records of patients with histologically proven Gb or SBM at our institution between November 2015 and September 2021 were reviewed to determine enrolment eligibility according to the inclusion and exclusion criteria. All the enrolled patients were classified based on the World Health Organization 2016 guidelines. The inclusion and exclusion criteria are shown in Supplemental Fig. 1. Overall, 109 patients met the study criteria and were divided into a training dataset (December 23, 2015, and October 9, 2019 [*n* = 76]) and a time-independent validation dataset (October 18, 2019, to September 26, 2021 [*n* = 33]). Demographic and clinical data are summarized in Table 1.

**Fig. 1:**
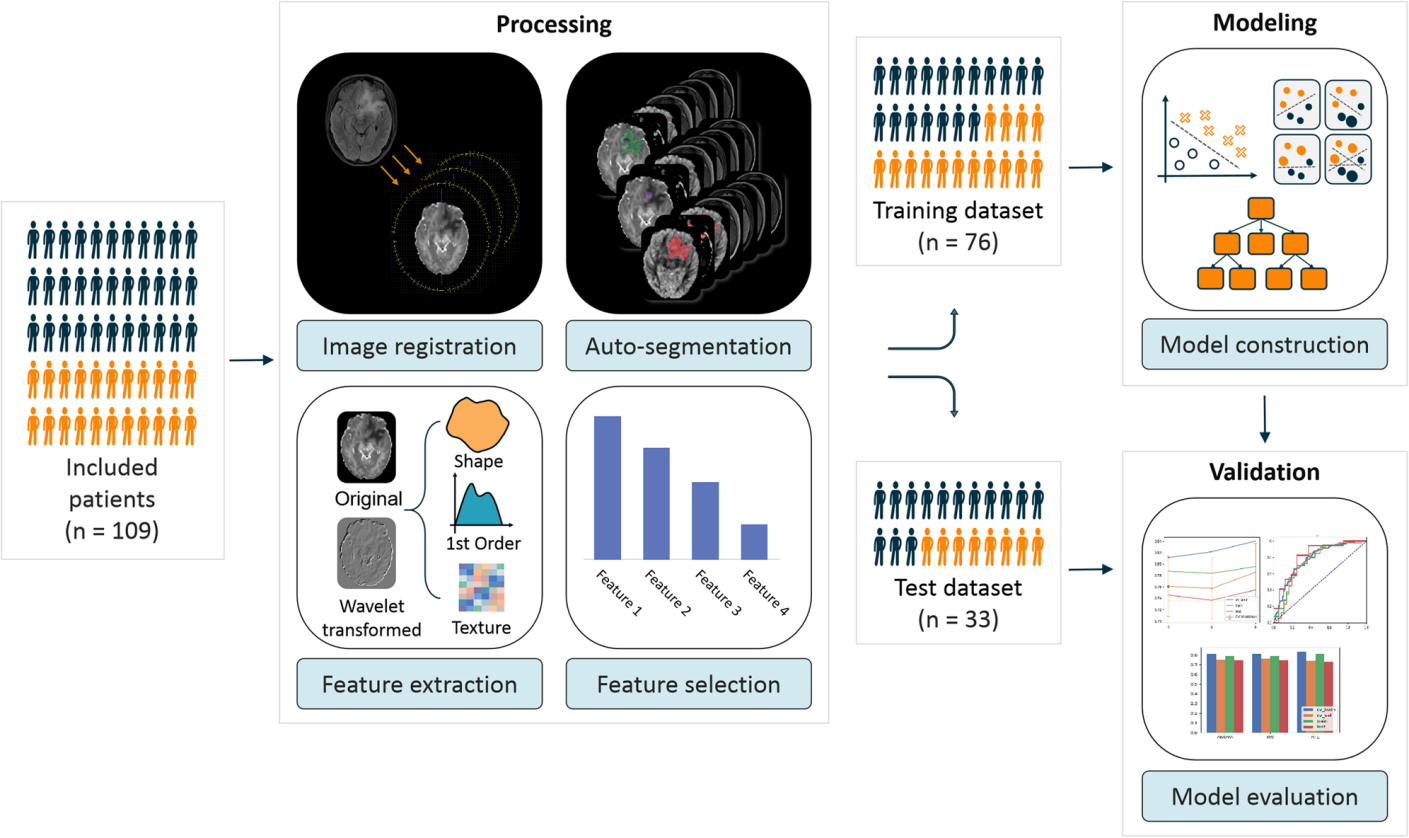
Radiomics workflow

**Table 1:** Clinical characteristics of the patients in the training and test datasets

Characteristic	Training dataset (*n* = 76)			Validation dataset (*n* = 33)			*p* value
	Gb (*n* = 40)	SBM (*n* = 36)	*p* value	Gb (*n* = 17)	SBM (*n* =16)	*p* value	
Age, years							
Mean ± SD	53.0 ± 10.0	52.9 ± 11.5	0.973	55.4 ± 10.7	58.7 ± 9.9	0.369	0.069
Gender,* n*			0.299			0.226	0.994
Male (%)	22 (55.0)	24 (66.7)		12 (70.6)	8 (50.0)		
Female (%)	18 (45.0)	12 (33.3)		5 (29.4)	8 (50.0)		
Variety of SBM,* n*							
Lung,* n*							
Adenocarcinoma (%)		25 (69.4)			13 (81.1)		
Squamous cell carcinoma (%)		1 (2.8)					
Neuroendocrine carcinoma (%)		4 (11.1)			1 (6.3)		
Small cell lung carcinoma (%)		1 (2.8)					
Poorly differentiated carcinoma (%)		1 (2.8)					
Stomach,* n*							
Adenocarcinoma (%)		1 (2.8)			0		
Kidney,* n*							
Clear cell carcinoma (%)		1 (2.8)			1 (6.3)		
Uterus,* n*							
Endometrial carcinoma (%)		0			1 (6.3)		
Unknown site,* n* (%)		2 (5.5)					

*Gb*, glioblastoma; *SBM*, single brain metastasis; *SD*, standard deviation

### Sample size and radiomics number estimation

According to the events per predictor variable and thumb rules, 10–15 samples are required for each predictor variable to yield a stable estimate [26, 29]. For the power calculation of the validation dataset, > 11 patients were required to acquire 80% power and a type I error rate of 5% [30]. Our dataset included 109 patients, of whom 76 and 33 were categorized into the training and validation datasets, respectively, meeting the sample size requirement. Specifically, in the training dataset, the minimum sample size of one tumor type was 36; thus, the maximum number of features included in the radiomic model construction was 4.

### Image acquisition

MR acquisitions were performed using a 3.0-T MRI scanner (MAGNETOM Prisma; Siemens Healthcare, Erlangen, Germany) with a 64-channel head and neck integrated coil. MR data included morphological MRI sequences (T2-weighted image [T2WI], fluid-attenuated inversion recovery [FLAIR], T1-weighted image [T1WI], three-dimensional contrast-enhanced T1 magnetization prepared rapid gradient echo [CE-T1 MPRAGE]) and diffusion MRI. Diffusion MRI was performed using six different *b*-values (0, 500, 1000, 1500, 2000, and 2500 s/mm^2^) and every nonzero *b*-value in 30 encoding directions [31]. CE-T1 MPRAGE was acquired after intravenous injection of 0.2 mL/kg gadopentetate dimeglumine (Magnevist, Bayer Schering Pharma AG, Berlin, Germany) using a high-pressure syringe, followed by a 20-mL saline flush at the same injection rate. CE-T1 MPRAGE images were obtained after contrast agent administration and reconstructed into 20 axial slices before use. All MRI sequence parameters are listed in Supplemental Table 1. NODDI parametric maps (including ICVF, ISOVF, and ODI), apparent diffusion coefficient (ADC), and diffusion tensor imaging (DTI) parametric maps (including AD, FA, MD, and RD) were calculated from the multi-*b*-value diffusion MRI data using in-house-developed post-processing software, NeuDiLab, based on the open-resource tool DIPY Toolbox (http://dipy.org).

### Process of radiomics analysis

The radiomics analysis of NODDI based on multiple VOIs was briefly structured into three parts: processing, modeling, and validation (Fig. 1).

### Image registration and segmentation

**Fig. 2:**
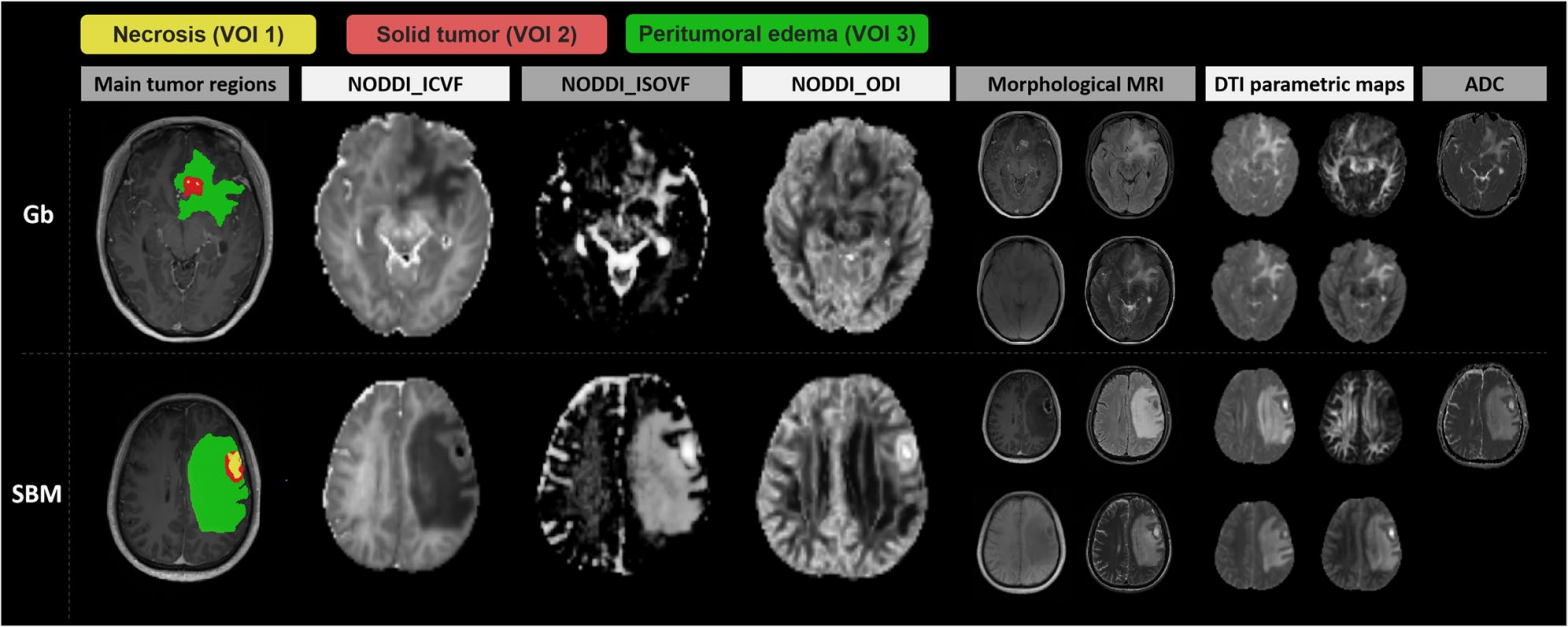
Necrosis (VOI 1), solid tumor (VOI 2), and peritumoral edema (VOI 3) are indicated by the yellow, red, and green lines, respectively

All morphological MR images, NODDI parametric maps, and ADC and DTI parametric maps were registered to FLAIR images using the open-source software ITK-SNAP (version 3.8.0, http://www.itksnap.org). A detailed description is provided in Supplemental Material E1. The multiple VOIs are defined as main tumor regions, including necrosis, solid tumor, and peritumoral edema areas. The VOIs on main tumor regions were delineated by automatic segmentation. Specifically, the nnU-Net trained by BraTs 2020 Challenge data was used to segment lesions automatically [32]. Then, the segmentations were discussed and revised by two radiologists (J.B. and X.M. with 5 and 10 years of experience, respectively), and the consensus results were used as the ground truth for segmentation. Examples of the two final segmentation cases based on automatic segmentation are shown in Fig. 2.

### Feature extraction

Features were extracted from the main tumor regions using the open-source software FeAture Explorer (FAE, version 0.5.2) [33], the backend of which was based on PyRadiomics (version 3.0). Overall, there were 851 radiomics features, including 14 shape features, 18 first-order features, and 75 textural features extracted from each of the original NODDI parametric maps and the eight sub-bands of its wavelet transformation. The extracted textural features included those based on the (1) gray-level co-occurrence matrix, (2) gray-level dependence matrix, (3) gray-level run-length matrix, (4) gray-level size zone matrix, and (5) neighborhood gray-tone difference matrix.

### Feature reduction and selection

After feature extraction, all radiomics feature values were normalized between 0 and 1 according to the min-max method. In feature reduction, the Pearson correlation coefficient (PCC) for each pair of features was calculated, and if the PCC of the feature pair was higher than 0.90, only one of the features was randomly retained. Further feature selection was performed based on analysis of variance, relief, and recursive feature elimination.

### Model construction

Finally, radiomics models were constructed using logistic regression and support vector machine with a linear kernel. For each tumor region, distinct radiomics models were individually constructed based on the NODDI parameter maps (ICVF, ISOVF, and ODI), as well as their combined application, to discern between Gb and SBM. Furthermore, a series of radiomics models were constructed using ADC, a composite of four morphological MRI sequences (T2WI, T1WI, FLAIR, and CE-T1 MPRAGE), and DTI (AD, FA, MD, and RD) for comparative analysis. To determine the hyper-parameter of each model, five-fold cross-validation was applied to the training dataset. After determining the hyper-parameter, all training data were retrained for the final models. The final models were evaluated using the time-independent validation dataset and determined by the best performance on the time-independent validation dataset. FAE was used for model training and pipeline operations for feature reduction and selection.

### Model evaluation

Receiver operating characteristic (ROC) analysis was employed to illustrate model performance, sensitivity, specificity, accuracy, and area under the receiver operating characteristic curve (AUC), and their 95% confidence intervals (CIs) were calculated for quantitative evaluation.

### Statistical analyses

The statistical analyses were performed using SPSS (version 21.0) and MedCalc (version 20.015). Differences in clinical characteristics between Gb and SBM were assessed by the chi-squared test and independent samples *t*-test, as appropriate. DeLong’s test was performed to observe the difference in the AUCs for the different models. All statistical tests assessed on significance according to a two-tailed threshold of *p* < 0.05.

## Results

### Patients’ clinical characteristics

The clinical characteristics of the patients in the study datasets are provided in Table 1. There were no significant differences between the training and validation datasets in terms of clinical characteristics (all *p* > 0.05).

In total, 57 (52.2%) Gbs and 52 (47.8%) SBMs were included. In the training and validation datasets, the Gb rates were 52.6% (40/76) and 51.5% (17/33), respectively, with no significant difference (*p* = 0.915).

Neurite orientation dispersion and density imaging radiomics models based on multiple volumes of interest

The predictive performance of the constructed prediction models, utilizing NODDI parameter maps and their combination, varies across different main tumor regions. Generally, the performance of the single-parameter NODDI model is inferior to that of the combined NODDI model. Specifically, in the necrosis region, the AUCs of models for the three NODDI parameter maps ranged from 0.621 to 0.676, whereas the combined model showed slightly higher discrimination ability, with an AUC of 0.701 (95% CI, 0.541–0.860). In the solid tumor region, the AUCs of models for the three NODDI parameter maps ranged from 0.790 to 0.901, and among all models, it was observed that the combined model exhibited superior discriminative power with an AUC of 0.904 (95% CI, 0.789–1.000). Within the peritumoral edema region, the AUCs of models for a single NODDI parameter map ranged from 0.713 to 0.812; however, even when integrated into a combined model, only moderate discrimination ability was achieved, with an AUC of 0.820 (95% CI, 0.664–0.976).

**Fig. 3:**
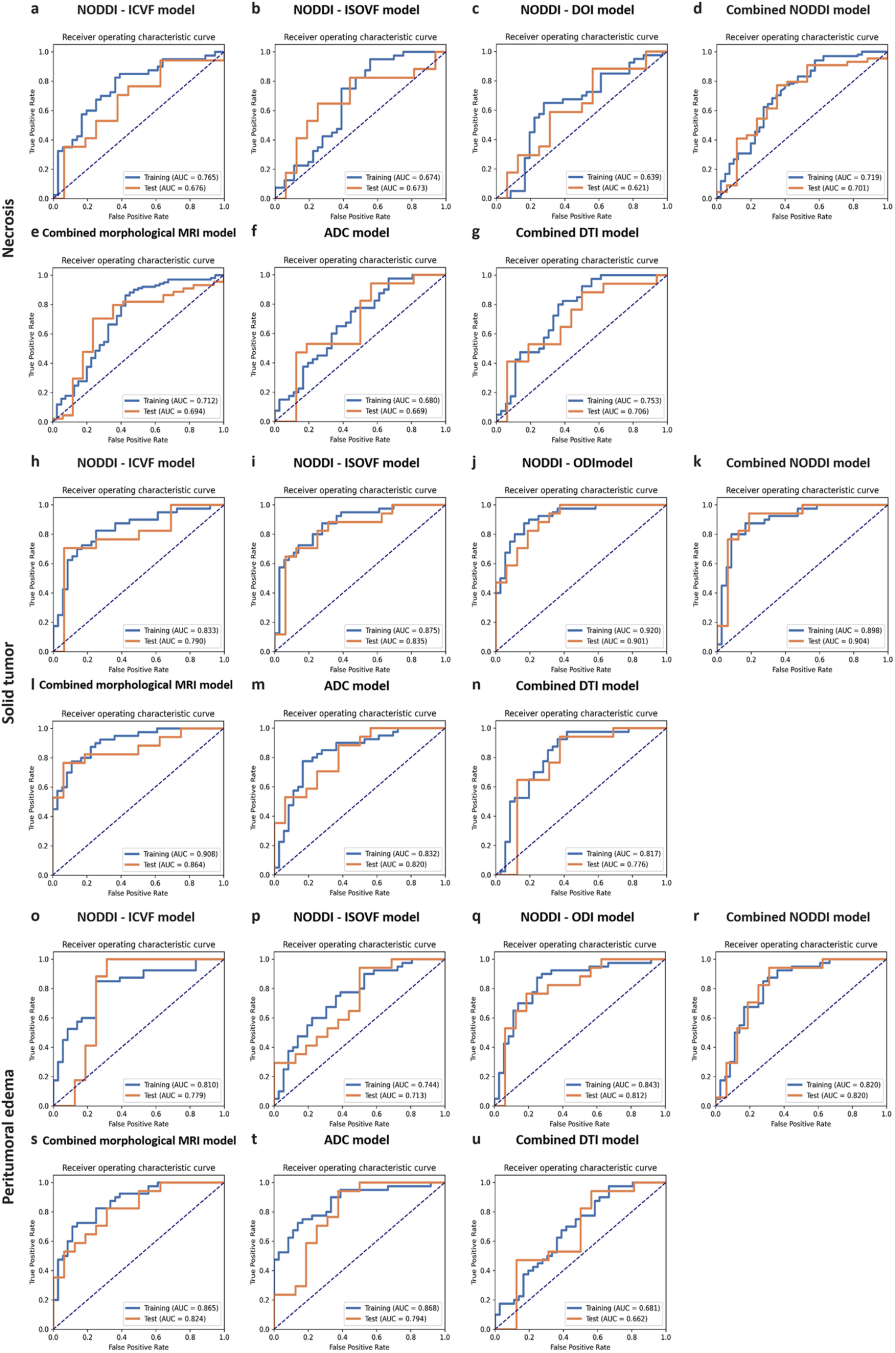
Receiver operating characteristic curves for different radiomics models based on the main tumor regions (**a**–**u**). Necrosis (**a**–**g**); solid tumor (**h**–**n**); peritumoral edema (**o**–**u**)

**Fig. 4:**
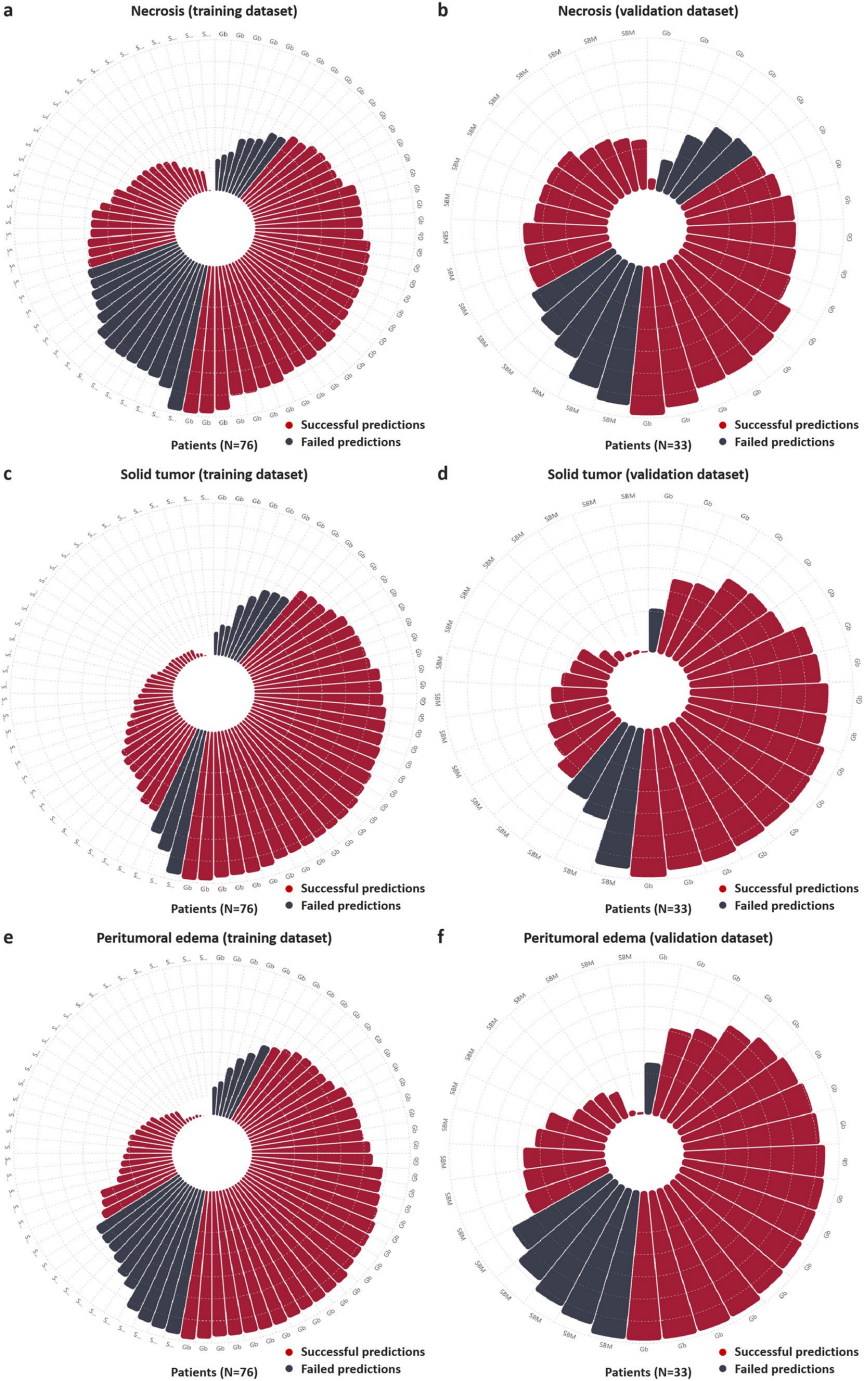
Rose plots depicting the predictive performance for different neurite orientation dispersion and density imaging radiomics models based on the main tumor regions (**a**–**f**). The red bar with the predicted probability value indicates the successful predictions of the model in the training dataset; the gray bar with the predicted value indicates the failed predictions of the model in the training dataset. The same is applicable for the validation dataset

**Fig. 5:**
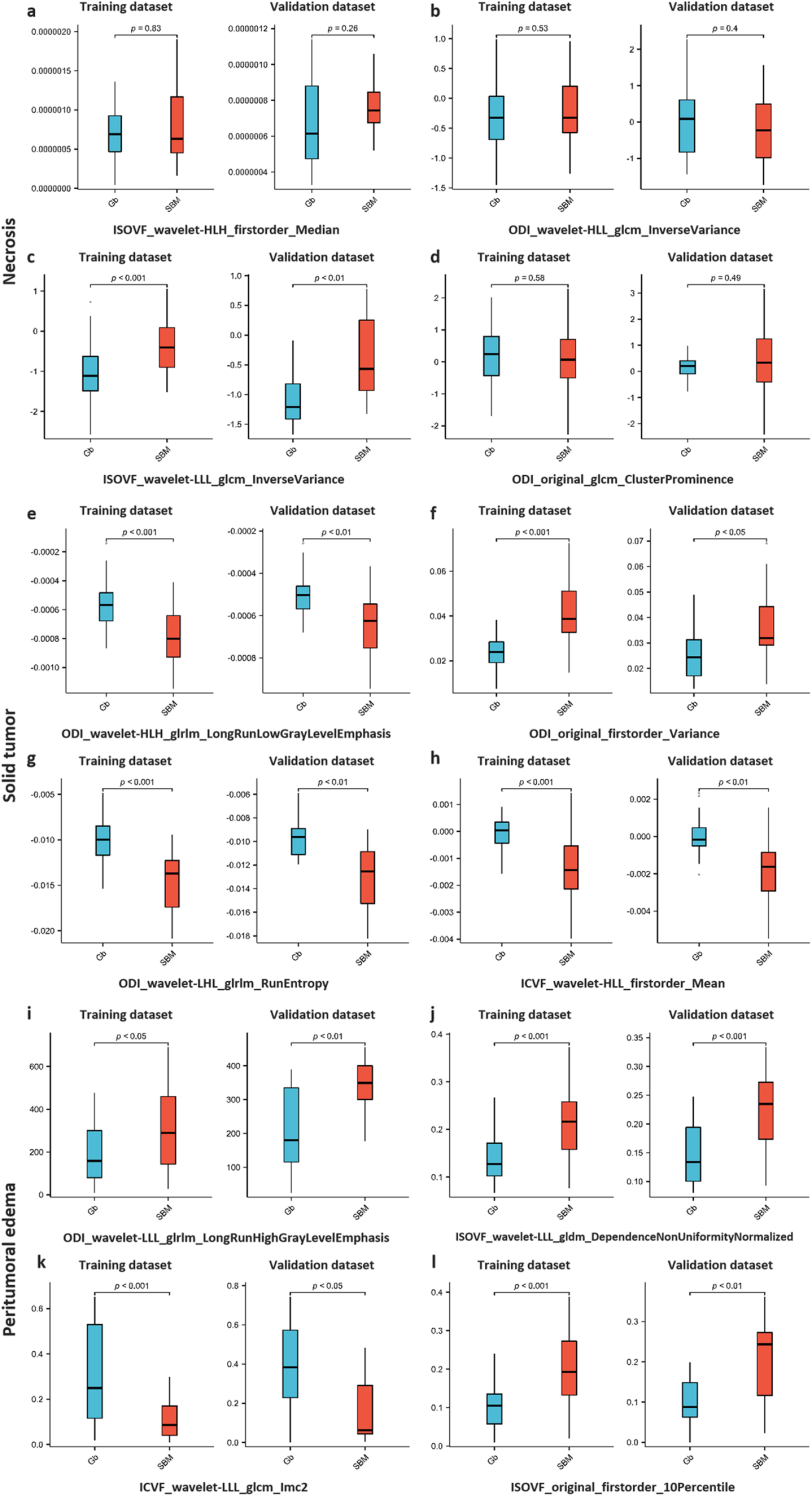
Box plot of the four selected features distinguishing between glioblastoma (Gb) and solitary brain metastasis (SBM). Necrosis (**a**–**d**); solid tumor (**e**–**h**); peritumoral edema (**i**–**l**)

The results of additional evaluation indicators are presented in Table 2. Figure 3 displays the ROC curves of different models in three main tumor regions. The prediction results of the training and validation datasets of the combined NODDI model are illustrated in Fig. 4. The differential evaluation of the four selected features between Gb and SBM is shown in Fig. 5. Additionally, the details of the radiomics process, such as the pairing parameters of feature selection methods and classifiers, are shown in Supplemental Figure 2, whereas the selected feature of different models is shown in Supplemental Table 2. Considering the superior discriminatory ability exhibited by the combined NODDI model within the solid tumor region, the DeLong test results for this model and other model are provided in Supplemental Table 3. However, no significant differences were observed in most of the results.

**Table 2:** The performance of validation dataset for NODDI radiomics, morphological MRI sequence, ADC, and DTI models

Main tumor regions	Model	Sensitivity	Specificity	Accuracy	AUC (95% CI)
Necrosis	NODDI - ICVF model	0.705	0.625	0.666	0.676 (0.485–0.867)
	NODDI - ISOVF model	0.647	0.750	0.697	0.673 (0.477–0.868)
	NODDI - DOI model	0.882	0.437	0.666	0.621 (0.422–0.820)
	Combined NODDI model	0.772	0.647	0.737	0.701 (0.541–0.860)
	Combined morphological MRI model	0.704	0.764	0.721	0.694 (0.531–0.856)
	ADC model	0.941	0.437	0.697	0.669 (0.472–0.866)
	Combined DTI model	0.884	0.500	0.697	0.706 (0.521–0.890)
Solid tumor	NODDI - ICVF model	0.705	0.937	0.818	0.790 (0.621–0.959)
	NODDI - ISOVF model	0.647	0.937	0.787	0.835 (0.688–0.980)
	NODDI - DOI model	0.823	0.812	0.818	0.901 (0.799–1.000)
	Combined NODDI model	0.942	0.812	0.878	0.904 (0.789–1.000)
	Combined morphological MRI model	0.764	0.937	0.848	0.864 (0.734–0.993)
	ADC model	0.882	0.625	0.757	0.820 (0.678–0.961)
	Combined DTI model	0.941	0.625	0.787	0.776 (0.596–0.954)
Peritumoral edema	NODDI - ICVF model	1.000	0.687	0.848	0.779 (0.587–0.971)
	NODDI - ISOVF model	0.941	0.500	0.727	0.713 (0.533–0.892)
	NODDI - DOI model	0.764	0.812	0.787	0.812 (0.656–0.968)
	Combined NODDI model	0.941	0.6875	0.818	0.820 (0.664–0.976)
	Combined morphological MRI model	0.824	0.688	0.758	0.824 (0.684–0.964)
	ADC model	0.941	0.625	0.787	0.794 (0.632–0.955)
	Combined DTI model	0.941	0.437	0.697	0.662 (0.464–0.859)

*95% CI*, 95% confidence interval

### Performance comparison

Lateral comparison involved the utilization of combined morphological MRI, ADC, and combined DTI models. Within the necrosis region, these models exhibited comparable discrimination ability to the NODDI model, showcasing a general discriminative capacity with AUC values ranging from 0.669 to 0.706. Within the solid tumor region, the combined morphological MRI, ADC, and combined DTI models achieved AUCs of 0.864, 0.820, and 0.776, respectively. Although the combined morphological MRI model exhibited acceptable discriminatory ability, it showed some disparity compared with the NODDI model’s performance; indeed, as data volume or sample size significantly increased, the NODDI model displayed a distinct advantage in effectively identifying tumors. In terms of peritumoral edema regions, the combined morphological MRI (AUC = 0.824) and ADC (AUC = 0.794) models showed similar discrimination ability compared with the NODDI model. However, limited discriminatory capability was observed when using the combined DTI model in this context. To summarize our findings among all lateral compared models used here: When ADC alone or the combined DTI model is used, the discrimination ability of the model is not ideal. The combined morphological MRI model showed a similar differential ability to the combined NODDI model.

## Discussion

This study aimed to achieve accurate differentiation between Gb and SBM using preoperative MRI. Radiomics features were extracted from multiple VOIs based on NODDI, followed by construction of classification models. Through lateral comparison, we have showcased the significant potential of NODDI radiomics analysis, specifically within the solid tumor region for distinguishing between Gb and SBM.

Morphological MRI offers an initial examination of tumors for the identification of Gb and SBM; however, more than 40% of the cases were incorrectly classified using only morphological MRI, which remains a challenge in neuroradiology [9]. Radiomics analysis can extract visually imperceptible features and characterize the specificity of tumors, which can significantly improve the accuracy of tumor identification. Considering the effectiveness of radiomics, the selection of appropriate MRI technology is very important. We strongly recommend the combination of NODDI and radiomics. First, our results showed that DWI and DTI have limited ability to distinguish Gb and SBM and are considered appropriate as part of a multiparametric MRI protocol rather than as a single sequence to be combined with radiomics. A large meta-analysis also showed that DWI and DTI exhibited broad individual sensitivities and specificities and only a moderate diagnostic accuracy [34]. Unlike traditional diffusion MRI methods that offer limited information, NODDI provides a more nuanced characterization of brain tissue, including the density and orientation of neuronal fibers [16, 17, 35]. This is particularly useful in the study of neurological disorders, where subtle changes in tissue structure can be indicative of disease progression or response to therapy. As for morphological MRI, our results confirmed that morphological MRI had a similar performance to NODDI in differentiating Gb and SBM and did not show statistical difference, which may be related to the small sample size. NODDI provides a more detailed view of the brain’s microstructure compared with morphological MRI, offering advantages in specificity and biological significance. NODDI provides indices that relate more directly to the underlying microstructure of the brain’s white matter and enables the identification of tissue subtypes and related injuries with enhanced specificity in pathology, a capability that morphological MRI cannot offer. Thus, NODDI offers a means to relate diffusion MRI signals to tissue features via biophysically inspired modeling. This is particularly advantageous in clinical practice as it provides a basis for more biological explanations of tissue changes.

More attention should be paid to the biological characteristics of the main tumor region and its features. Compared with the other two main tumor regions, the NODDI radiomics model based on the solid tumor region demonstrates superior performance in differentiating between Gb and SBM. The differences between Gb and SBM in the solid tumor region are mainly reflected in the enhancement patterns [1, 36]. Gb is characterized by high vascularity, local hypoxia, abnormal angiogenesis, and inflammatory response. These factors lead to abnormal vascular permeability and disruption of the blood–brain barrier in the solid region of Gb, resulting in irregular and intense enhancement patterns on MRI. The enhancement patterns of SBM may vary depending on the characteristics of the primary cancer site and metastatic lesions. The enhancement in SBM is primarily attributed to the disruption of the blood–brain barrier at the metastatic site. Generally, compared with Gb, SBM tends to have clear boundaries and a more homogeneous enhancement pattern. The selected features include two texture features and two first-order features. The textural features describe the texture variations in the ODI parameter map under wavelet analysis, quantifying the degree of texture complexity and capturing the irregularity of the enhancement region. The first-order features describe the mean and variance of the VOI, recording changes in signal intensity in the enhancement region. Moreover, previous studies have demonstrated that changes in the ODI signal can correspond to changes in the microglia number, morphology, and activation state in various disease models [37]. The role of microglia in Gb and SBM is not the same [38]. In this study, three selected features of the ODI signal features demonstrated significant differences between Gb and SBM. This difference may indicate potential differences in the microglia or other cellular and molecular components in the solid tumor region of Gb and SBM.

Gb and SBM involve distinct molecular mechanisms and pathways in the formation of peritumoral edema [39–41]. Gb primarily induces edema through infiltrative growth, potentially facilitating its invasive growth by actively modifying the extracellular matrix and aiding infiltrative spread within the brain. Contrastingly, SBM typically results in vasogenic edema due to metastatic cancer cells compromising the integrity of the blood–brain barrier at new sites, leading to structural and functional impairments of the vascular wall, thus causing local vascular leakage and edema. Furthermore, SBMs may carry and release angiogenic factors unique to the primary tumor, further exacerbating the blood–brain barrier breakdown and edema development. In our study, the NODDI radiomics model for the peritumoral edema region only demonstrated moderate discriminative ability. This result is inconsistent with a previous study [17], and might be because of the limited capability of the selected features to distinguish between infiltrative edema and vasogenic edema.

The effectiveness of differentiating Gb from SBM through the NODDI model in the necrotic region is unsatisfactory. This inadequacy is likely due to the similar malignant biological characteristics of the two tumor types. The necrotic regions in both Gb and SBM result from their rapid and disorganized growth, leading to insufficient blood supply and eventual tumor tissue necrosis [42, 43]. Compared with Gb, the necrosis in SBM may be more associated with the characteristics of the primary tumor. For example, melanoma, breast cancer, and lung cancer may carry specific genes and proteins that promote rapid growth when metastasizing to the brain, accelerating tumor growth, and leading to rapid vascular insufficiency and subsequent necrosis. The imaging features of these specific genes and proteins may either not be captured or have lacked sufficient specificity. This can also be concluded from the fact that most of the Gb and SBM features were not statistically different.

The repeatability of radiomics is one of the main constraints of implementing models in clinical practice. Sharing of clinical data and radiomics models can make it easier to replicate all radiomics studies (including NODDI radiomics). However, data sharing may be hindered by patient privacy issues, and model sharing is constrained by the disclosure of modeling processes and platforms (or code). The code compatibility is uncertain; the majority of these do not work. Fortunately, some open-source radiomics software, such as LIFEx [44] and FAE, provide necessary data processing and analysis functions, and can be easily used. The FAE was used in this study, including for the visualization of results and sharing of models, thus potentially reducing the need for retraining radiomics at a new site before implementation.

The limitations of this study must be acknowledged. First, our model was trained and validated using retrospective, small-sample data collected from a single institution. Second, more sequences or imaging modalities, such as perfusion-weighted imaging, should be added for comparison to improve persuasiveness. Finally, more refined subregions should be considered to explore the relationship between image features and VOIs.

In conclusion, the NODDI radiomics model showed promising performance for preoperative discrimination between Gb and SBM, and radiomics features can be incorporated into the multidimensional phenotypic features that describe tumor heterogeneity.

## Supplementary Information

Below is the link to the electronic supplementary material. 

Supplementary file1 (PDF 434 KB)

## Abbreviations

ADC: Apparent diffusion coefficient
AUC: Area under the receiver operating characteristic curve
CE-T1 MPRAGE: Contrast-enhanced T1 magnetization prepared rapid gradient echo
CI: Confidence interval
DTI: Diffusion tensor imaging
DWI: Diffusion-weighted imaging
FLAIR: Fluid-attenuated inversion recovery
Gb: Glioblastoma
ICVF: Intracellular volume fraction
ISOVF: Isotropic volume fraction
MRI: Magnetic resonance imaging
NODDI: Neurite orientation dispersion and density imaging
ODI: Orientation dispersion index
PCC: Pearson correlation coefficient
ROC: Receiver operating characteristic
ROI: Region of interest
SBM: Solitary brain metastasis
T1WI: T1-weighted image
T2WI: T2-weighted image
VEC: Extracellular volume fraction
VOI: Volume of interest

## Declarations

### Guarantor

The scientific guarantor of this publication is Guohua Zhao.

### Statistics and biometry

No complex statistical methods were necessary for this paper.

### Study subjects or cohorts overlap

Some study subjects have been previously reported in 31st Annual Meeting of ISMRM, London, UK, 2022, ID.3062.

### Methodology

- retrospective
- case-control study
- performed at one institution
