# Supplementary material for: High-performance presurgical differentiation of glioblastoma and metastasis by means of multiparametric neurite orientation dispersion and density imaging (NODDI) radiomics
Source: Eur Radiol. 2024 Mar 15;34(10):6616–28. doi: 10.1007/s00330-024-10686-8 (PMC11399163; doi:10.1007/s00330-024-10686-8)
Supplement: Supplementary file 1 — Supplementary file1 (PDF 434 KB) [file 330_2024_10686_MOESM1_ESM.pdf]

High-performance presurgical differentiation of glioblastoma and metastasis by means of multiparametric neurite orientation dispersion and density imaging (NODDI) radiomics

ELECTRONIC SUPPLEMENTARY MATERIAL

Supplemental Figures

Fig. 1

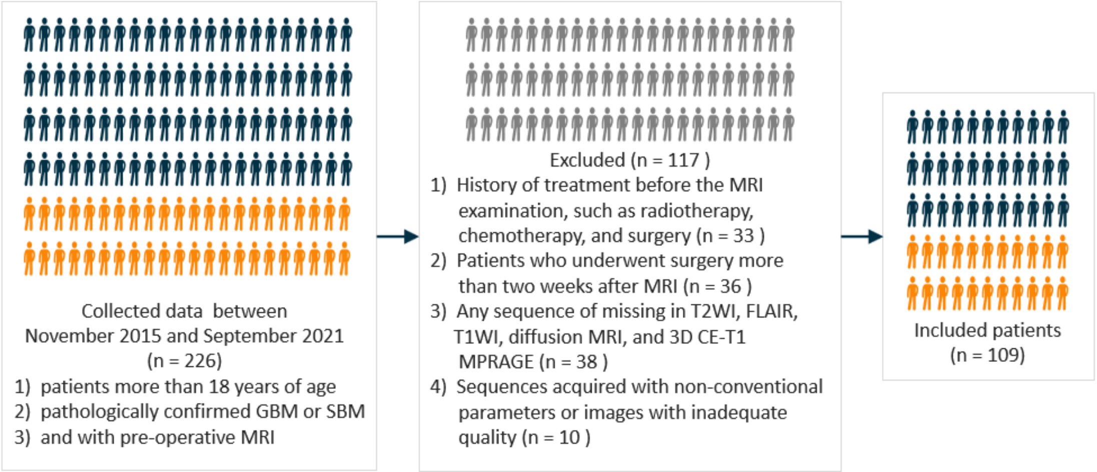

Fig. 1: The inclusion and exclusion criteria for the radiomics analysis. T2WI, T2-weighted image; 3D CE-T1 MPRAGE, three-dimensional contrast-enhanced T1 magnetization prepared rapid gradient echo.

Fig. 2

a

NODDI - ICVF (Necrosis)

Training<br>Validation	ANOVA	Relief	RFE
LR	0.762<br>0.585	0.651<br>0.401	0.765<br>0.676
SVM	0.757<br>0.559	0.644<br>0.360	0.755<br>0.665

NODDI - ICVF (Solid tumor)

Training<br>Validation	ANOVA	Relief	RFE
LR	0.833<br>0.772	0.837<br>0.574	0.889<br>0.599
SVM	0.833<br>0.790	0.843<br>0.585	0.853<br>0.664

NODDI - ICVF (Peritumoral edema)

Training<br>Validation	ANOVA	Relief	RFE
LR	0.819<br>0.772	0.746<br>0.772	0.858<br>0.688
SVM	0.810<br>0.779	0.728<br>0.759	0.856<br>0.699

b

NODDI - ISOVF (Necrosis)

Training<br>Validation	ANOVA	Relief	RFE
LR	0.873<br>0.669	0.674<br>0.673	0.917<br>0.643
SVM	0.872<br>0.665	0.672<br>0.662	0.910<br>0.647

NODDI - ISOVF (Solid tumor)

Training<br>Validation	ANOVA	Relief	RFE
LR	0.892<br>0.822	0.875<br>0.835	0.818<br>0.817
SVM	0.845<br>0.831	0.903<br>0.816	0.815<br>0.810

NODDI - ISOVF (Peritumoral edema)

Training<br>Validation	ANOVA	Relief	RFE
LR	0.873<br>0.669	0.674<br>0.673	0.918<br>0.643
SVM	0.872<br>0.665	0.690<br>0.670	0.910<br>0.647

c

NODDI - ODI (Necrosis)

Training<br>Validation	ANOVA	Relief	RFE
LR	0.828<br>0.570	0.626<br>0.620	0.872<br>0.592
SVM	0.827<br>0.588	0.639<br>0.621	0.867<br>0.588

NODDI - ODI (Solid tumor)

Training<br>Validation	ANOVA	Relief	RFE
LR	0.922<br>0.871	0.830<br>0.496	0.920<br>0.901
SVM	0.914<br>0.897	0.811<br>0.610	0.910<br>0.900

NODDI -ODI (Peritumoral edema)

Training<br>Validation	ANOVA	Relief	RFE
LR	0.828<br>0.610	0.693<br>0.717	0.843<br>0.812
SVM	0.831<br>0.607	0.684<br>0.713	0.840<br>0.811

d

Combined NODDI (Necrosis)

Training<br>Validation	ANOVA	Relief	RFE
LR	0.873<br>0.669	0.721<br>0.634	0.917<br>0.643
SVM	0.872<br>0.665	0.719<br>0.701	0.910<br>0.647

Combined NODDI (Solid tumor)

Training<br>Validation	ANOVA	Relief	RFE
LR	0.901<br>0.846	0.902<br>0.868	0.817<br>0.824
SVM	0.890<br>0.857	0.898<br>0.904	0.790<br>0.794

Combined NODDI (Peritumoral edema)

Training<br>Validation	ANOVA	Relief	RFE
LR	0.763<br>0.779	0.760<br>0.809	0.820<br>0.820
SVM	0.711<br>0.824	0.758<br>0.816	0.819<br>0.815

e

Combined conventional MRI (Necrosis)

Training<br>Validation	ANOVA	Relief	RFE
LR	0.845<br>0.714	0.712<br>0.694	0.843<br>0.592
SVM	0.847<br>0.702	0.719<br>0.688	0.839<br>0.588

Combined conventional MRI (Solid tumor)

Training<br>Validation	ANOVA	Relief	RFE
LR	0.901<br>0.816	0.576<br>0.540	0.908<br>0.864
SVM	0.899<br>0.809	0.410<br>0.474	0.903<br>0.852

Combined conventional MRI (Peritumoral edema)

Training<br>Validation	ANOVA	Relief	RFE
LR	0.792<br>0.816	0.865<br>0.824	0.720<br>0.768
SVM	0.785<br>0.805	0.801<br>0.798	0.717<br>0.746

f

ADC (Necrosis)

Training<br>Validation	ANOVA	Relief	RFE
LR	0.820<br>0.632	0.680<br>0.669	0.830<br>0.632
SVM	0.826<br>0.621	0.663<br>0.680	0.818<br>0.632

ADC (Solid tumor)

Training<br>Validation	ANOVA	Relief	RFE
LR	0.835<br>0.816	0.620<br>0.776	0.839<br>0.801
SVM	0.832<br>0.820	0.625<br>0.776	0.839<br>0.801

ADC (Peritumoral edema)

Training<br>Validation	ANOVA	Relief	RFE
LR	0.917<br>0.768	0.868<br>0.794	0.920<br>0.735
SVM	0.907<br>0.746	0.877<br>0.779	0.901<br>0.743

g

Combined DTI (Necrosis)

Training<br>Validation	ANOVA	Relief	RFE
LR	0.764<br>0.570	0.753<br>0.706	0.763<br>0.573
SVM	0.750<br>0.574	0.767<br>0.664	0.774<br>0.599

Combined DTI (Solid tumor)

Training<br>Validation	ANOVA	Relief	RFE
LR	0.850<br>0.596	0.817<br>0.776	0.891<br>0.621
SVM	0.849<br>0.588	0.794<br>0.765	0.872<br>0.603

Combined DTI (Peritumoral edema)

Training<br>Validation	ANOVA	Relief	RFE
LR	0.819<br>0.632	0.681<br>0.662	0.830<br>0.629
SVM	0.818<br>0.618	0.633<br>0.622	0.819<br>0.636

Fig. 2: Matrices **a-g** showed the performance (AUC) of the radiomics models constructed with different pairing parameters. The orange squares represented the best performance, and the dark gray squares represented models constructed with paired parameters that obtain a high AUC, but the results showed a tendency for overfitting

Supplemental Tables

Table 1 Sequence parameters

Sequences	Slice orientation	TR/TE (ms)	Number of slices	Slice thickness	FOV (mm^2)	Acquisition matrix	Scan time
T1WI	Axial	250.0/2.46	20	5.0 mm	220×220	314×314	37 s
T2WI	Axial	4,090.0/99.0	20	5.0 mm	220×220	733×733	34 s
FLAIR	Axial	8,000.0/81.0	20	5.0 mm	220×220	314×314	1 min 38 s
Multi-b-value diffusion MRI	Axial	2,500.0/71.0	60	2.2 mm	220×220	100×100	6 min 34 s
CE-T1 MPRAGE	Sagittal	2,300.0/2.32	176	0.9 mm	240×240	266×266	5 min 21 s

CE-T1 MPRAGE, contrast-enhanced T1 magnetization prepared rapid gradient echo.

Table 2. The selected features on different main tumor regions and sequences

Tumor regions and sequences	Radiomics features	Coefficient in model
(Necrosis) NODDI - ICVF model	ICVF_wavelet-HHL_firstorder_InterquartileRange	-0.692
	ICVF_wavelet-LLH_firstorder_InterquartileRange	0
	ICVF_wavelet-LLH_firstorder_RobustMeanAbsoluteDeviation	-0.180
	ICVF_wavelet-LLL_glcm_Idn	0.519
(Necrosis) NODDI - ISOVF model	ISOVF_wavelet-HHH_glcm_ClusterShade	0.688
	ISOVF_original_firstorder_Minimum	-1.091
	ISOVF_wavelet-HLH_firstorder_Median	-0.396
	ISOVF_wavelet-LLL_glcm_InverseVariance	0
(Necrosis) DOI model	ODI_wavelet-LHL_glcm_InverseVariance	0.310
	ODI_wavelet-HLL_glcm_InverseVariance	-0.210
	ODI_wavelet-LLL_glrlm_ShortRunHighGrayLevelEmphasis	-0.156
	ODI_original_glcm_ClusterProminence	-0.685
(Necrosis) Combined NODDI model	ISOVF_wavelet-HLH_firstorder_Median	-1.465
	ODI_wavelet-HLL_glcm_InverseVariance	-0.063
	ODI_original_glcm_ClusterProminence	-0.825
	ISOVF_wavelet-LLL_glcm_InverseVariance	-0.159
(Necrosis) Combined morphological MRI model	CET1_wavelet-HHH_firstorder_Variance	-0.704
	FLAIR_wavelet-HHH_firstorder_Skewness	-0.126
	CET1_wavelet-HLL_firstorder_Skewness	-0.624
	CET1_wavelet-HHH_firstorder_Skewness	-0.257
(Necrosis) ADC model	ADC_wavelet-HHH_firstorder_10Percentile	0.325
	ADC_wavelet-LHH_firstorder_Variance	-0.705
	ADC_wavelet-HHH_glcm_ClusterShade	0.377
	ADC_wavelet-HHH_firstorder_TotalEnergy	0.191
(Necrosis) Combined DTI model	DTI_FA_wavelet-LLH_ngtdm_Complexity	0.969
	DTI_MD_wavelet-HLH_firstorder_90Percentile	-5.624
	DTI_AD_wavelet-HHL_firstorder_10Percentile	4.099
	DTI_MD_wavelet-LLH_firstorder_Mean	0.370
(Solid tumor) NODDI - ICVF model	ICVF_original_shape_SurfaceArea	2.471
	ICVF_wavelet-HLL_firstorder_Mean	1.576
	ICVF_wavelet-LHL_firstorder_10Percentile	1.796
	ICVF_wavelet-LHL_firstorder_Mean	1.117
(Solid tumor) NODDI - ISOVF model	ISOVF_original_firstorder_Variance	-4.683
	ISOVF_wavelet-HLL_firstorder_Median	0
	ISOVF_wavelet-LHL_firstorder_10Percentile	2.461
	ISOVF_wavelet-LHL_firstorder_Mean	0.319
(Solid tumor) DOI model	ODI_original_firstorder_Variance	-6.696
	ODI_wavelet-HLH_glrlm_LongRunLowGrayLevelEmphasis	5.041
	ODI_wavelet-LHL_firstorder_10Percentile	5.783
	ODI_wavelet-LHL_glrlm_RunEntropy	2.473
(Solid tumor) Combined	ODI_wavelet-HLH_glrlm_LongRunLowGrayLevelEmphasis	1.533

NODDI model	ODI_wavelet-LHL_glrlm_RunEntropy	-2.263
	ICVF_wavelet-HLL_firstorder_Mean	2.002
	ODI_original_firstorder_Variance	1.597
(Solid tumor) Combined morphological MRI model	CET1_wavelet-HLH_firstorder_10Percentile	-0.879
	CET1_wavelet-HLH_firstorder_Energy	1.586
	CET1_wavelet-LLH_glrlm_LongRunHighGrayLevelEmphasis	-1.251
	Flair_original_glrlm_GrayLevelVariance	-0.781
(Solid tumor) ADC model	ADC_original_glcm_Idmn	-0.683
	ADC_original_glcm_InverseVariance	6.766
	ADC_original_ngtdm_Complexity	-0.839
	ADC_original_ngtdm_Contrast	-2.708
(Solid tumor) Combined DTI model	DTI_FA_original_glrlm_LongRunLowGrayLevelEmphasis	-6.645
	DTI_FA_wavelet-LLL_ngtdm_Complexity	-5.371
	DTI_RD_wavelet-LHL_firstorder_Mean	-3.005
	DTI_AD_original_shape_Flatness	2.346
(Peritumoral edema) NODDI - ICVF model	ICVF_original_shape_Sphericity	-0.538
	ICVF_original_shape_SurfaceVolumeRatio	0.215
	ICVF_wavelet-LLL_glcm_Imc2	0.691
	ICVF_wavelet-LLH_glcm_Autocorrelation	0.548
(Peritumoral edema) NODDI - ISOVF model	ISOVF_wavelet-LLL_gldm_DependenceNonUniformityNormalized	-1.094
	ISOVF_wavelet-HLH_glrlm_ShortRunLowGrayLevelEmphasis	0.529
	ISOVF_original_firstorder_10Percentile	-0.683
	ISOVF_wavelet-LHH_glrlm_ShortRunLowGrayLevelEmphasis	0.294
(Peritumoral edema) DOI model	ODI_original_shape_Elongation	0.290
	ODI_original_shape_Sphericity	-0.642
	ODI_wavelet-LHL_firstorder_InterquartileRange	-1.185
	ODI_wavelet-LLL_glrlm_LongRunHighGrayLevelEmphasis	0
(Peritumoral edema) Combined NODDI model	ODI_wavelet-LLL_glrlm_LongRunHighGrayLevelEmphasis	-2.635
	ISOVF_wavelet-LLL_gldm_DependenceNonUniformityNormalized	-3.878
	ICVF_wavelet-LLL_glcm_Imc2	-6.835
	ISOVF_original_firstorder_10Percentile	-1.571
(Peritumoral edema) Combined morphological MRI model	T2_wavelet-LHL_glrlm_RunEntropy	10.164
	CET1_wavelet-HLL_firstorder_MeanAbsoluteDeviation	3.659
	CET1_wavelet-HLL_glcm_Imc2	9.896
	CET1_wavelet-HLL_gldm_DependenceEntropy	-4.808
(Peritumoral edema) ADC model	ADC_wavelet-LHH_glcm_Correlation	-3.280
	ADC_wavelet-LLH_glszm_SmallAreaLowGrayLevelEmphasis	1.394
	ADC_wavelet-LHH_firstorder_Median	-3.187
	ADC_wavelet-HLH_firstorder_Mean	0
(Peritumoral edema) Combined DTI model	DTI_RD_wavelet-HHH_firstorder_10Percentile	2.023
	DTI_FA_wavelet-LHH_firstorder_Variance	-2.856
	DTI_MD_wavelet-HHH_glcm_ClusterShade	3.887
	DTI_FA_wavelet-HHH_firstorder_TotalEnergy	1.779

Note: The formulas and detailed descriptions of the features are available on the pyradiomics website (https://pyradiomics.readthedocs.io/en/stable/features.html).

Table 3. The DeLong test result in the validation datasets

Index	Model 1 vs. Model 2	*p*-value
1	(Solid tumor) Combined NODDI vs. (Solid tumor) NODDI-ICVF	0.284
2	(Solid tumor) Combined NODDI vs. (Solid tumor) NODDI-ISOVF	0.106
3	(Solid tumor) Combined NODDI vs. (Solid tumor) NODDI-DOI	0.927
4	(Solid tumor) Combined NODDI vs. (Solid tumor) Combined morphological MRI	0.683
5	(Solid tumor) Combined NODDI vs. (Solid tumor) ADC	0.358
6	(Solid tumor) Combined NODDI vs. (Solid tumor) Combined DTI	0.228
7	(Solid tumor) Combined NODDI vs. (Necrosis) Combined NODDI	0.083
8	(Solid tumor) Combined NODDI vs. (Necrosis) NODDI-ICVF	0.052
9	(Solid tumor) Combined NODDI vs. (Necrosis) NODDI-ISOVF	0.057
10	(Solid tumor) Combined NODDI vs. (Necrosis) NODDI-DOI	**0.015**
11	(Solid tumor) Combined NODDI vs. (Necrosis) Combined morphological MRI	0.071
12	(Solid tumor) Combined NODDI vs. (Necrosis) ADC	**0.017**
13	(Solid tumor) Combined NODDI vs. (Necrosis) Combined DTI	0.094
14	(Solid tumor) Combined NODDI vs. (Peritumoral edema) Combined NODDI	0.344
15	(Solid tumor) Combined NODDI vs. (Peritumoral edema) NODDI-ICVF	0.3132
16	(Solid tumor) Combined NODDI vs. (Peritumoral edema) NODDI-ISOVF	0.109
17	(Solid tumor) Combined NODDI vs. (Peritumoral edema) NODDI-DOI	0.381
18	(Solid tumor) Combined NODDI vs. (Peritumoral edema) Combined morphological MRI	0.217
19	(Solid tumor) Combined NODDI vs. (Peritumoral edema) ADC	0.261
20	(Solid tumor) Combined NODDI vs. (Peritumoral edema) Combined DTI	0.009

Supplemental Appendix

E1: Image registration

ITK-snap provided the registration function for multi-modal images. Specifically, FLAIR images were used as the registration master, and the NODDI parametric maps were used as moving images. We use the automatic registration function for image registration, which is based on a rigid transformation model. A mutual information method was used to measure image similarity. The multi-resolution parameters were set as follows: coarsest level, 2x; finest level, 1x. Finally, linear interpolation was used to create registered images.

T2WI, T1WI, and CE-T1 MPRAGE were registered to FLAIR images using the same method. It should be emphasized that the images of each participant were registered before manual and automatic ROI segmentation. Segmentation masks affect morphological MR images and NODDI parametric maps.

Eur Radiol (2024) Bai J, He MY, Gao E, et al.
